# Prediction of symptom development and aortic valve replacement in patients with low-gradient severe aortic stenosis

**DOI:** 10.1093/ehjopen/oeae018

**Published:** 2024-03-06

**Authors:** Daisuke Miyahara, Masaki Izumo, Yukio Sato, Tatsuro Shoji, Risako Murata, Ryutaro Oda, Taishi Okuno, Shingo Kuwata, Yoshihiro J Akashi

**Affiliations:** Department of Cardiology, St Marianna University School of Medicine, 2-16-1, Sugao, Miyamae-ku, Kawasaki 216-8511, Japan; Department of Cardiology, St Marianna University School of Medicine, 2-16-1, Sugao, Miyamae-ku, Kawasaki 216-8511, Japan; Department of Cardiology, St Marianna University School of Medicine, 2-16-1, Sugao, Miyamae-ku, Kawasaki 216-8511, Japan; Department of Cardiology, St Marianna University School of Medicine, 2-16-1, Sugao, Miyamae-ku, Kawasaki 216-8511, Japan; Department of Cardiology, St Marianna University School of Medicine, 2-16-1, Sugao, Miyamae-ku, Kawasaki 216-8511, Japan; Department of Cardiology, St Marianna University School of Medicine, 2-16-1, Sugao, Miyamae-ku, Kawasaki 216-8511, Japan; Department of Cardiology, St Marianna University School of Medicine, 2-16-1, Sugao, Miyamae-ku, Kawasaki 216-8511, Japan; Department of Cardiology, St Marianna University School of Medicine, 2-16-1, Sugao, Miyamae-ku, Kawasaki 216-8511, Japan; Department of Cardiology, St Marianna University School of Medicine, 2-16-1, Sugao, Miyamae-ku, Kawasaki 216-8511, Japan

**Keywords:** Aortic stenosis, Low gradient, Exercise stress echocardiography, *E/e*′

## Abstract

**Aims:**

Current evidence on the prognostic value of exercise stress echocardiography (ESE) in asymptomatic patients with low-gradient severe aortic stenosis (AS) is limited. Therefore, this study aimed to elucidate its prognostic implications for patients with low-gradient severe AS and determine the added value of ESE in risk stratification for this population.

**Methods and results:**

This retrospective observational study included 122 consecutive asymptomatic patients with either moderate [mean pressure gradient (MPG) < 40 mmHg and aortic valve area (AVA) 1.0–1.5 cm^2^] or low-gradient severe (MPG < 40 mmHg and AVA < 1.0 cm^2^) AS and preserved left ventricular ejection fraction (≥50%) who underwent ESE. All patients were followed up for AS-related events. Of 143 patients, 21 who met any exclusion criteria, including early interventions, were excluded, and 122 conservatively managed patients [76.5 (71.0–80.3) years; 48.3% male] were included in this study. During a median follow-up period of 989 (578–1571) days, 64 patients experienced AS-related events. Patients with low-gradient severe AS had significantly lower event-free survival rates than those with moderate AS (log-rank test, *P* < 0.001). Multivariable Cox regression analysis showed that the mitral *E*/*e*′ ratio during exercise was independently associated with AS-related events (hazard ratio = 1.075, *P* < 0.001) in patients with low-gradient severe AS.

**Conclusion:**

This study suggests that asymptomatic patients with low-gradient severe AS have worse prognoses than those with moderate AS. Additionally, the mitral *E*/*e*′ ratio during exercise is a useful parameter for risk stratification in patients with low-gradient severe AS.

## Graphical Abstract

**Graphical Abstract:**
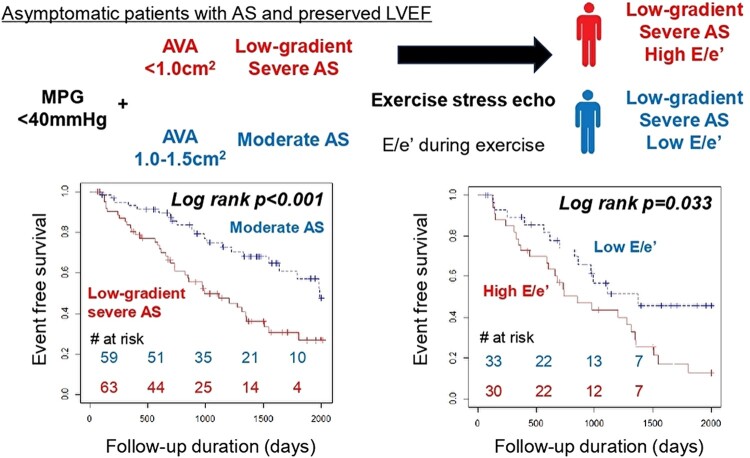

## Introduction

Aortic stenosis (AS) is a prevalent valvular heart disease, particularly in developing countries, and its incidence increases with an aging population,^1,2^ affecting 2–9% of older persons.^2–4^ Echocardiography serves as the cornerstone of evaluating and grading AS. Aortic stenosis severity is generally determined based on aortic valve area (AVA) and mean pressure gradient (MPG). Discordant echocardiographic measurements are occasionally encountered; some patients may have severe stenosis based on the AVA but not severe stenosis based on MPG, even when left ventricular ejection fraction (LVEF) is preserved.^5^

Previous studies have indicated that patients with low-flow, low-gradient, severe AS represent a subgroup with advanced-stage AS, reduced stroke volume (SV), and poor prognoses.^6–9^ Conversely, another study indicated that the prognoses of these patients were similar to those of patients with moderate AS.^10^ Symptomatic patients with paradoxical low-flow low-gradient (PLFLG) severe AS have been reported to have poor prognoses in many studies, and guidelines recommend invasive therapy for stage D valvular heart disease.^11^ However, this inconsistency between MPG and AVA is common in daily clinical practice, making proper classification of AS severity challenging. Therefore, the prognostic implications of low-gradient severe AS remain controversial and are not fully understood, and most previous studies have relied on echocardiographic measurements performed at rest.

Exercise testing has demonstrated the utility of identifying symptoms or abnormal haemodynamic responses during exercise in patients with asymptomatic AS. Exercise stress echocardiography (ESE) provides additional information on the flow and pressure dynamics associated with AS. However, limited evidence supports ESE as a valuable tool for evaluating asymptomatic AS. Previous studies have reported that increased MPG levels during exercise and exercise-induced pulmonary hypertension (EIPH) were important indicators of symptom development and adverse outcomes.^12,13^ However, other studies have questioned the efficacy of these findings in risk stratification, possibly because they are influenced by contractile reserves and myocardial responses.^14^ Therefore, this study aimed to elucidate the prognoses of asymptomatic patients with low-gradient severe AS in comparison with those with moderate AS and explore additional information ESE can offer for risk stratification in this cohort.

## Methods

### Study design

This study retrospectively observed 143 consecutive asymptomatic patients with either moderate- (MPG < 40 mmHg and AVA 1.0–1.5 cm^2^) or low-gradient severe (MPG < 40 mmHg and AVA < 1.0 cm^2^) AS and preserved LVEF (≥50%), who underwent ESE between January 2013 and December 2021 at St Marianna University Hospital. All patients in this study are at least 20 years old. The study protocol was approved by the ethics committee of St Marianna University School of Medicine (No. 6250), and the need for informed consent was waived due to the retrospective nature of the study.

### Resting echocardiography

All patients underwent comprehensive two-dimensional and Doppler transthoracic echocardiography before exercise testing in accordance with the American Society of Echocardiography guidelines.^15^ An experienced sonographer performed all echocardiographic procedures using Vivid E9 or E95 ultrasound systems (General Electric Healthcare, Little Chalfont, UK). The left ventricular (LV) end-diastolic volume (EDV) and end-systolic volume (ESV) were measured using the Simpson biplane method. The LVEF was calculated as follows: [(EDV − ESV)/EDV] × 100. Continuous-wave Doppler was used to measure the maximal aortic valve velocities in apical three- or five-chamber views, and the peak and mean gradients were estimated based on the simplified Bernoulli equation. The LV outflow tract (LVOT) diameter was measured using zoomed parasternal long-axis views. The LVOT velocities acquired using pulsed-wave Doppler and the velocity–time integrals (VTI LVOT) were measured.

The SV was calculated using the following formula: (LVOT diameter/2)^2^ × 3.14 × VTI LVOT. Subsequently, the SV index (SVi) and cardiac output (CO) were estimated using the following formulas: SVi = SV/body surface area (BSA) and CO = SV × heart rate (HR). The AVA was calculated using the continuity equation, and the AVA index was calculated by dividing it with the BSA. The global LV afterload was estimated using the valvulo-arterial impedance (Zva), formulated as follows: Zva = (systolic blood pressure + MPG)/SVi.^16^ The systolic pulmonary artery pressure (SPAP) was derived from the regurgitant jet of tricuspid regurgitation (TRPG), adding the estimated right atrial pressure from the inferior vena cava (IVC). The tricuspid annulus plane systolic excursion (TAPSE) was assessed using the M-mode on the tricuspid annulus and expressed as the longitudinal systolic function of shortening the right ventricle. The *e*′ was measured from the apical four-chamber view, with a 2–5 mm sample volume placed at the septal corner of the mitral annulus. Absolute values of echocardiographic measurements during exercise stress were used and not the change from rest. To avoid difficulties in measuring E and A waves during exercise, data before fusion at an HR of around 100 b.p.m. were adopted. In atrial fibrillation cases, data obtained at a relatively constant RR interval were used.

#### Exercise stress echocardiography

Following comprehensive transthoracic echocardiography at rest, the patients underwent a symptom-limited graded exercise test in a semi-supine position on a bicycle ergometer table tilted to 20°, as previously described.^17^ After maintaining an initial workload of 10 W for 3 min, the workload was increased by 10 W every 3  min. A single-lead electrocardiogram was continuously monitored, and blood pressure was measured at rest and every 1 min during exercise. Patients were excluded if they had the following abnormalities: (i) occurrence of angina, dizziness, or syncope; (ii) a decrease in systolic blood pressure (SBP) below baseline and (iii) complex arrhythmia during exercise.

### Endpoint

Follow-up data were collected from the medical records. The primary endpoint of the present study was the occurrence time of the first composite endpoint, defined as cardiovascular death, aortic valve replacement due to AS-related symptoms (syncope, dyspnoea, and angina), and hospitalization for heart failure.

### Statistical analysis

Continuous variables are expressed as mean values with standard deviations, or median values with interquartile ranges (IQR). Categorical variables are expressed as numbers and percentages. Student’s *t*-test was used to analyse continuous variables with normal distributions, and the Mann–Whitney *U*-test was used to analyse continuous variables with non-normal distributions. Categorical variables were compared using Fisher’s exact or χ^2^ tests, as appropriate.

Statistical significance was set at *P* < 0.05. The cumulative probability of event-free survival was estimated using the Kaplan–Meier method and was compared between groups using a log-rank test. The optimal cut-off value of *E/e*′ during exercise was determined based on the receiver operating characteristics curve and the highest Youden index. Univariable and multivariable Cox proportional hazard models were used to calculate hazard ratios and 95% confidence intervals for clinical outcomes. Data analyses were performed using JMP 16 (SAS Institute Japan, Inc., Tokyo, Japan) and R statistical software (version 4.2.2; R Foundation for Statistical Computing, Vienna, Austria).

## Results

**Figure 1:**
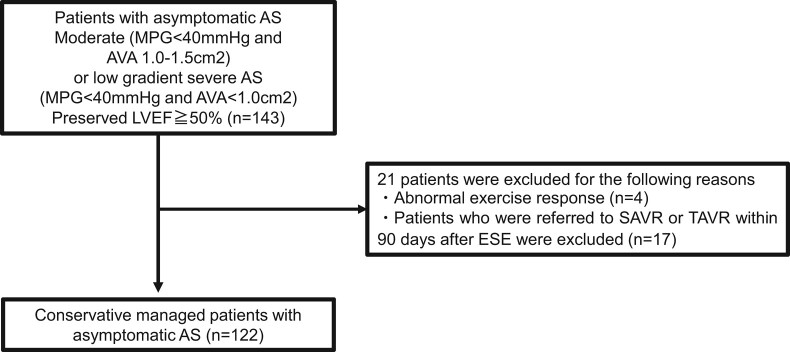
Flow chart showing study participant distribution.

A total of 21 patients were excluded due to the following reasons: (i) abnormal exercise response (*n* = 4; atrial fibrillation tachycardia, advanced atrioventricular block during exercise, ventricular tachycardia, and ST change) and (ii) early intervention [*n* = 17; transcatheter aortic valve replacement (TAVR) or surgical aortic valve replacement (SAVR) within 90 days after ESE]. The remaining 122 patients, who were managed conservatively, were included in this study (*Figure 1*).

### Baseline characteristics

*Table 1* shows the baseline characteristics of the study cohort. A total of 59 (48.3%) patients were male, and the median age was 76.5 (IQR: 71.0–80.3) years. No significant differences in sex, age, comorbidities, or medications were observed between patients with moderate AS and those with low-gradient severe AS. However, patients with low-gradient severe AS had significantly smaller BSAs.

**Table 1:** Baseline clinical characteristics of the population

	All (*n* = 122)	Moderate AS (*n* = 59)	LG severe AS (*n* = 63)	*P*-value
Sex, male	59 (48.3)	33 (55.9)	26 (41.3)	0.105
Age, years	76.5 (71.0–80.3)	76.0 (72.0–81.0)	77.0 (70.0–80.0)	0.912
Body surface area, m^2^	1.55 (1.42–1.71)	1.60 (1.50–1.80)	1.49 (1.39–1.63)	<0.001
*Hypertension*	80 (65.6)	41 (69.5)	39 (61.9)	0.378
Diabetes mellitus	24 (19.7)	9 (15.3)	15 (23.8)	0.235
Dyslipidaemia	52 (42.6)	27 (45.8)	25 (39.7)	0.497
CAD	24 (19.7)	12 (20.3)	12 (19.1)	0.858
Haemodialysis	5 (4.1)	2 (3.4)	3 (4.8)	0.703
Atrial fibrillation	29 (23.8)	16 (27.1)	13 (20.6)	0.401
β-Blocker	24 (19.7)	15 (25.4)	9 (14.3)	0.122
ACE inhibitor/ARB	48 (39.3)	24 (40.7)	24 (38.1)	0.770
MRA	4 (3.3)	2 (3.4)	2 (3.2)	0.947
Diuretics	10 (8.2)	6 (10.2)	4 (6.4)	0.442
Bicuspid	12 (9.9)	6 (10.2)	6 (9.5)	0.905

Data are mean ± SD, median (IQR), or *n* (%).

LG, low gradient; CAD, coronary artery disease; ACEi, angiotensin-converting enzyme inhibitor; ARB, angiotensin II receptor; MRA, mineralocorticoid receptor antagonist.

### Resting and exercise echocardiography

The ESE data are summarized in *Tables 2* and *3*. No significant differences in SBP or HR were observed between the two groups at rest. However, patients with low-gradient severe AS had significantly lower AVA and AVA indices, higher MPG, and smaller left ventricular end-diastolic volume and left ventricular end-systolic volume than those with moderate AS. The LVEF was similar in both groups. SV and CO were significantly smaller in patients with low-gradient severe AS, possibly because of the LV size. The percentage of low-flow status was significantly higher in patients with low-gradient severe AS. The two groups had no significant differences in diastolic function, right ventricular function, or SPAP. SBP during exercise was comparable between the two groups. However, patients with low-gradient severe AS had significantly higher HR than those with moderate AS. Patients with severe mitral regurgitation were not included in this study.

**Table 2:** Comparison of resting echocardiographic data between patients with moderate- and low-gradient severe aortic stenosis

	All (*n* = 122)	Moderate AS (*n* = 59)	LG severe AS (*n* = 63)	*P*-value
SBP, mmHg	140.1 (±21.2)	141.5 (±20.9)	138.9 (±21.5)	0.507
HR, b.p.m.	69.0 (62.0–77.9)	66.0 (62.0–73.0)	71.0 (62.0–82.0)	0.051
AVA, cm^2^	1.01 (±0.23)	1.20 (±0.14)	0.83 (±0.12)	<0.001
AVA index, cm^2^/m^2^	0.64 (±0.15)	0.74 (±0.13)	0.55 (±0.09)	<0.001
MPG, mmHg	20.5 (15.6–26.4)	16.2 (13.4–22.1)	24.4 (20.0–29.4)	<0.001
LVDd, mm	43.6 (±5.6)	44.6 (±6.0)	42.7 (±5.0)	0.051
LVDs, mm	26.0 (24.0–29.3)	27.0 (25.0–31.0)	26.0 (24.0–29.0)	0.058
LV mass index, g/m^2^	81.8 (73.1–98.3)	80.3 (71.1–97.3)	82.1 (73.9–98.5)	0.869
SV, mL	65.3 (58.0–74.1)	69.0 (61.0–81.1)	62.7 (54.0–72.0)	<0.001
SV index, mL/m^2^	42.5 (36.5–47.3)	43.4 (38.8–47.3)	41.5 (34.7–47.1)	0.084
Low flow, %	22 (18.0)	6 (10.2)	16 (25.4)	0.026
CO, L/min	4.6 (3.9–5.3)	4.9 (4.1–5.7)	4.2 (3.7–5.1)	0.027
LVEDV, mL	81.0 (67.6–99.0)	85.0 (73.0–103.9)	77.7 (67.0–94.0)	0.053
LVESV, mL	26.7 (22.0–34.0)	30.0 (22.5–39.5)	25.0 (21.0–31.4)	0.035
LVEF, %	66.1 (±6.0)	65.3 (±6.7)	66.7 (±5.2)	0.208
LAV index, mL/m^2^	37.5 (30.0–45.8)	37.4 (31.6–45.8)	37.6 (28.7–45.8)	0.877
E, cm/s	73.0 (59.5–95.0)	74.0 (58.0–94.0)	69.0 (60.0–98.0)	0.890
*e*′, cm/s	5.1 (4.3–6.1)	5.3 (4.5–6.1)	4.8 (4.1–6.0)	0.171
*E/e*′	13.7 (11.1–19.6)	13.4 (11.0–19.5)	14.8 (11.3–19.8)	0.358
*E/A*	0.74 (0.63–0.86)	0.78 (0.66–0.89)	0.73 (0.61–0.85)	0.715
Abnormal, %	78 (84.8)	36 (85.7)	42 (84.0)	
Pseudonormal, %	14 (15.2)	6 (14.3)	8 (16.0)	
ZVa, mmHg/mL/m^2^	3.78 (3.30–4.44)	3.68 (3.00–4.18)	4.12 (3.48–4.50)	0.010
MR ≥ 3°	1 (0.8)	0 (0)	1 (1.6)	0.331
TRPG, mmHg	23.0 (19.9–27.0)	22.3 (18.9–27.5)	23.5 (20.3–27.0)	0.828
SPAP, mmHg	26.0 (22.9–30.1)	25.3 (21.9–30.5)	26.5 (23.3–30.0)	0.838
TAPSE, mm	19.8 (16.9–22.1)	19.9 (17.7–23.0)	19.3 (16.7–21.5)	0.484

Data are mean ± SD, median (IQR), or *n* (%).

SBP, systolic blood pressure; LVDd, left ventricular diastolic dimension; LVDs, left ventricular systolic dimension; LAV, left atrial volume.

**Table 3:** Comparison of exercise echocardiographic data between the patients with moderate- and low-gradient severe aortic stenosis

	All (*n* = 122)	Moderate AS (*n* = 59)	LG severe AS (*n* = 63)	*P*-value
Exercise intensity, METs	3.5 (3.0–4.4)	3.5 (2.9–4.3)	3.7 (3.1–4.6)	0.357
Peak workload, W	40 (30–57.5)	40 (30–60)	40 (30–50)	0.200
SBP, mmHg	175.2 (±30.5)	172.7 (±28.1)	177.4 (±32.5)	0.409
HR, b.p.m.	110.3 (±21.2)	105.6 (±20.8)	114.4 (±20.9)	0.024
AVA, cm^2^	1.04 (0.92–1.25)	1.24 (1.14–1.41)	0.92 (0.79–1.02)	<0.001
AVA index, cm^2^/m^2^	0.66 (0.58–0.80)	0.77 (0.64–0.92)	0.59 (0.49–0.68)	<0.001
MPG, mmHg	26.4 (18.8–35.2)	20.0 (16.6–27.8)	31.6 (25.3–38.5)	<0.001
SV, mL	71.0 (61.3–83.2)	74.2 (68.9–88.9)	64.6 (55.4–78.0)	<0.001
SV index, mL/m^2^	46.3 (±10.6)	49.0 (±10.9)	43.8 (±9.8)	0.006
CO, L/min	7.8 (±1.9)	8.2 (±1.8)	7.5 (±1.8)	0.042
LVEDV, mL	86.0 (73.0–107.5)	90.0 (74.0–109.7)	81.4 (66.6–104.4)	0.079
LVESV, mL	24.0 (18.0–30.9)	25.0 (19.7–32.9)	22.7 (17.0–28.9)	0.330
LVEF, %	73.0 (68.5–76.0)	73.0 (67.0–76.0)	73.6 (69.0–75.9)	0.635
E, cm/s	127.0 (107.8–151.0)	125.0 (103.0–149.0)	131.0 (112.0–161.0)	0.133
*e*′, cm/s	8.1 (6.9–10.3)	8.0 (6.7–9.1)	8.3 (6.9–12.5)	0.011
*E/e*′	15.3 (11.8–18.8)	14.9 (12.7–19.3)	15.3 (10.3–18.0)	0.816
*E/A*	1.04 (0.85–1.23)	1.05 (0.83–1.24)	0.97 (0.85–1.22)	0.841
Abnormal relaxation, %	46 (50.6)	18 (43.9)	28 (56.0)	
Pseudonormal, %	45 (49.5)	23 (56.1)	22 (44.0)	
ZVa, mmHg/mL/m^2^	4.48 (3.71–5.27)	3.96 (3.29–4.91)	4.95 (4.09–5.71)	<0.001
TRPG, mmHg	45.4 (±11.6)	44.1 (±12.7)	46.5 (±10.5)	0.262
SPAP, mmHg	55.4 (±11.6)	54.1 (±12.7)	56.5 (±10.5)	0.262
Exercise-induced PH, %	45 (38.5)	22 (39.3)	23 (37.7)	0.861
TAPSE, mm	24.0 (±5.5)	24.4 (±5.6)	23.6 (±5.3)	0.421

Data are mean ± SD, median (IQR), or *n* (%).

MET, metabolic equivalent.

Aortic stenosis severity parameters during exercise, including AVA and AVA index, were significantly lower, and the MPG was higher in patients with low-gradient severe AS, consistent with the resting data. The LV size during exercise was not significantly different between the two groups; however, patients with low-gradient severe AS had significantly smaller SV and CO during exercise.

### Comparisons between outcomes in low-gradient severe aortic stenosis vs. moderate aortic stenosis

**Figure 2:**
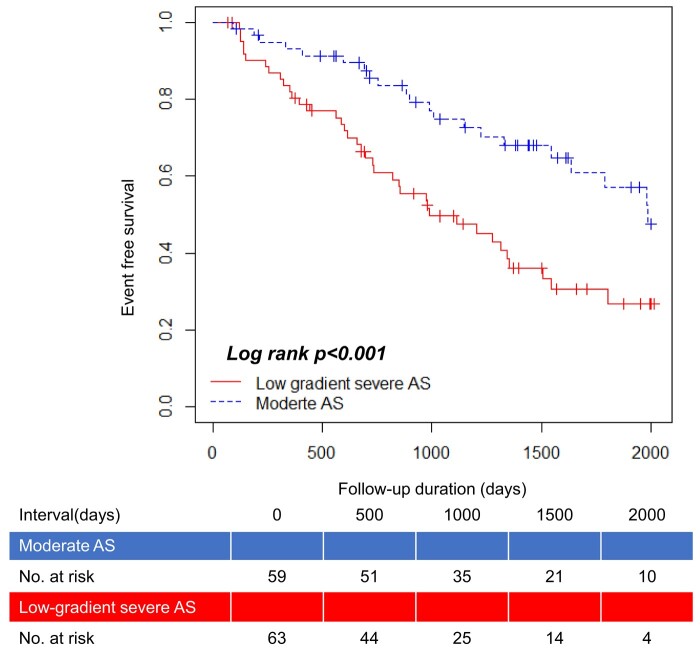
Event-free survival curve of moderate aortic stenosis vs. low-gradient severe aortic stenosis.

During a median follow-up period of 989 (IQR: 578–1571) days, the composite endpoint occurred in 64 patients (cardiovascular death, *n* = 2; hospitalization for heart failure, *n* = 2; and AVR, *n* = 58). The Kaplan–Meier analysis revealed a significantly lower event-free survival rate in patients with low-gradient severe AS than in those with moderate AS (log-rank test, *P* < 0.001; *Figure 2*). Multivariable Cox regression analysis revealed that low-gradient severe AS was independently associated with the event risk (hazard ratio = 2.386, *P* = 0.009 and *P* = 0.002; *Table 4*).

**Table 4:** Univariable and multivariable Cox regression analyses for predicting aortic valve-related events in patients with moderate and low-gradient severe aortic stenosis

	Univariable model		Multivariable model	
	Hazard ratio (95% CI)	*P*-value	Hazard ratio (95% CI)	*P*-value
Age, years	1.032 (1.002–1.063)	0.039	1.038 (1.007–1.071)	0.018
SBP, mmHg	0.998 (0.986–1.010)	0.715	0.994 (0.981–1.007)	0.386
Sex, male	1.077 (0.655–1.770)	0.771	1.052 (0.592–1.867)	0.863
HR, b.p.m.	1.018 (0.999–1.038)	0.064	1.010 (0.988–1.032)	0.398
Low-gradient severe AS	2.344 (1.397–3.933)	0.001	2.386 (1.367–4.166)	0.002

### Univariable and multivariable Cox regression analyses

The prognostic factors in patients with low-gradient severe AS were investigated. The cut-off value for the mitral *E/e*′ index during exercise was set at 15.4 (receiver operating characteristic curve, *P* = 0.007; area under the curve (AUC), 0.749; sensitivity, 65.9%; specificity, 86.3%). The high mitral *E*/*e*′ index during exercise (≥15.4) was significantly associated with an increased risk of adverse events in the univariable analysis, in patients with low-gradient severe AS (*P* = 0.011; *Table 5*). Contrastingly, the SPAP at rest and during exercise, and low-flow status were not associated with the event risk.

**Table 5:** Univariable and multivariable Cox regression analyses for predicting aortic valve-related events in patients with low-gradient severe aortic stenosis

	Univariable model		Age-, sex-, and BSA-adjusted model		Model 1		Model 2	
	Hazard ratio (95% CI)	*P*-value	Hazard ratio (95% CI)	*P*-value	Hazard ratio (95% CI)	*P*-value	Hazard ratio (95% CI)	*P*-value
AVA at rest, cm^2^	0.277 (0.033–2.996)	0.262	0.041 (0.003–0.638)	0.021	0.037 (0.002–0.623)	0.021		
MPG at rest, mmHg	1.022 (0.973–1.072)	0.377	1.025 (0.976–1.074)	0.304			1.023 (0.975–1.072)	0.340
*E/e*′ at rest (continuous)	1.034 (0.987–1.078)	0.136	1.003 (0.984–1.076)	0.193				
Low flow at rest	1.065 (0.528–2.148)	0.860						
SPAP at rest, mmHg	1.040 (0.981–1.102)	0.182						
ZVa at rest, mmHg/mL/m^2^	1.018 (0.789–1.296)	0.891						
AVA during exercise, cm^2^	0.624 (0.141–2.863)	0.541	0.394 (0.066–2.221)	0.299				
MPG during exercise, mmHg	1.004 (0.984–1.022)	0.657	1.001 (0.980–1.020)	0.885				
*E/e*′ during exercise	1.060 (1.026–1.091)	<0.001	1.065 (1.028–1.099)	<0.001	1.075 (1.035–1.113)	<0.001	1.066 (1.031–1.102)	<0.001
High *E/e*′ during exercise	2.312 (1.209–4.419)	0.011	2.340 (1.187–4.614)	0.014	2.337 (1.187–4.600)	0.014	2.350 (1.192–4.635)	0.014
SPAP during exercise, mmHg	1.040 (0.981–1.102)	0.182						
ZVa during exercise	0.863 (0.680–1.064)	0.195						
mmHg/mL/m^2^								

Model 1 was adjusted for age, sex, BSA, AVA at rest, and the high *E/e*′ ratio during exercise.

Model 2 was adjusted for age, sex, BSA, MPG at rest, and the high *E/e*′ ratio during exercise.

BSA, body surface area; CI, confidence interval.

After adjusting for age, sex, and BSA, the AVA at rest and the high mitral *E/e*′ during exercise were significantly associated with an increased risk of adverse events. The variables for the multivariable Cox regression analysis were selected (Model 1: age, sex, BSA, AVA at rest, and the high mitral *E/e*′ index during exercise; Model 2: age, sex, BSA, MPG at rest, and the high mitral *E/e*′ index during exercise). Multivariable Cox regression analysis revealed that the AVA at rest and the high mitral *E/e*′ index during exercise were independently associated with the event risk (hazard ratio = 0.024 and 2.350, *P* = 0.009 and *P* = 0.014, respectively; *Table 5*). *E/e*′ during exercise was also significantly associated with adverse events in flow-specific analyses [low-flow: HR 1.075 (1.012–1.142); normal-flow: HR 1.056 (1.012–1.093); both *P* < 0.05].

### Outcomes associated with the high mitral *E/e*′ index during exercise in patients with low-gradient severe aortic stenosis

**Figure 3:**
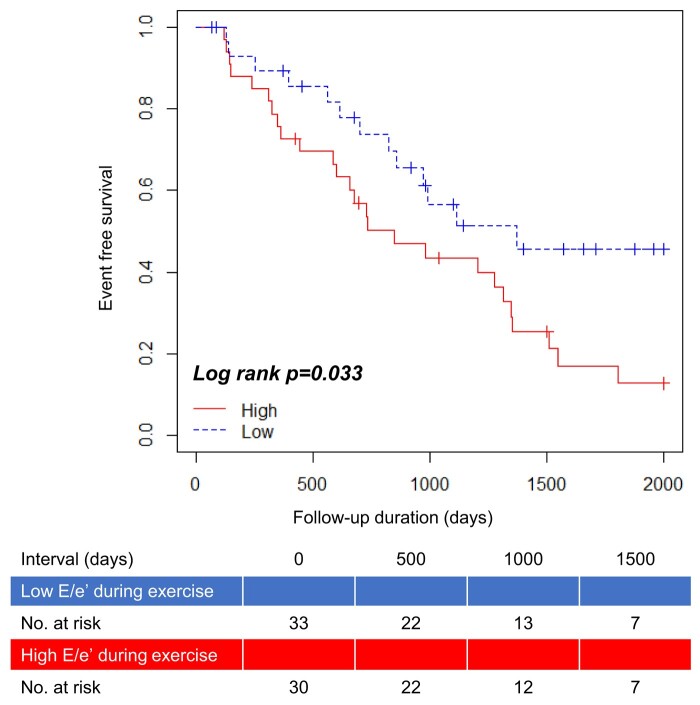
Event-free survival curve according to mitral *E/e*′ in patients with low-gradient severe AS.

**Figure 4:**
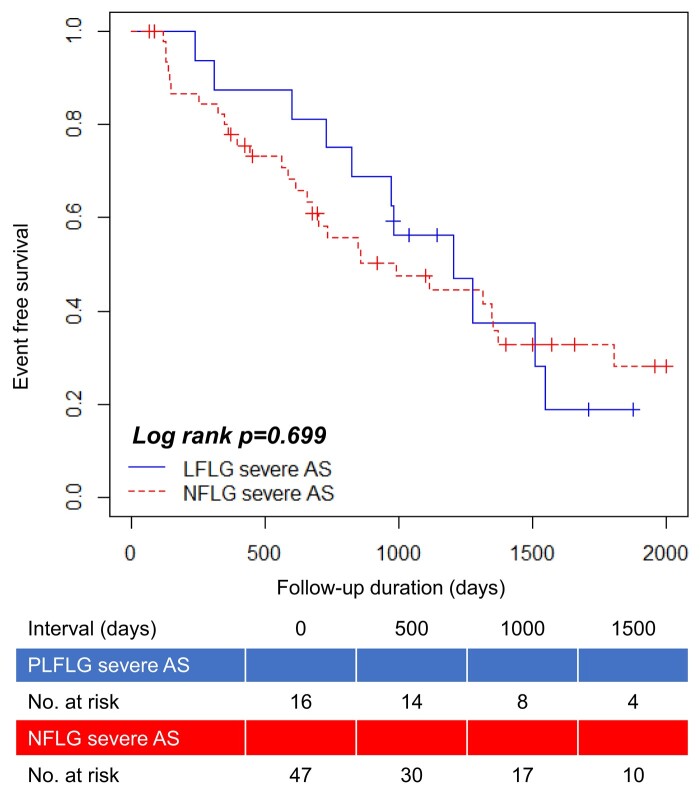
Event-free survival curve of reduced vs. preserved stroke volume index in patients. PLFLG, paradoxical low-flow low gradient; NFLG, normal-flow low gradient.

The patients in this study were divided into two groups based on their mitral *E*/*e*′ during exercise as follows: low *E/e*′ (<15.4, *n* = 33) and high *E*/*e*′ (≥15.4, *n* = 30) groups. *Table 6* summarizes the ESE data. No significant differences in sex, age, BSA, and the percentage of low-flow status and MR (≥3°) were observed between patients in the low and high *E/e*′ groups. The high *E/e*′ group had a significantly larger left atrial size, lower LVEF at rest, and lower AVA and AVA index during exercise than the low *E/e*′ group. Kaplan–Meier analysis indicated that the high *E/e*′ group had a significantly lower event-free survival rate than the low *E/e*′ group (log-rank test, *P* = 0.009; *Figure 3*). Prognostic stratification according to the mitral *E/e*′ index during exercise was possible in patients with low-gradient severe AS. Kaplan–Meier analysis stratified according to reduced (≤35 mL/m^2^, *n* = 47) and preserved (>35 mL/m^2^, *n* = 16) SVi showed no statistically significant difference in the rate of event-free survival (log-rank, *P* = 0.860; *Figure 4*).

**Table 6:** Comparison of resting and exercise echocardiographic findings between the low and high *E/e*′ during exercise in patients with low-gradient severe AS

	Low *E/e*′ (*n* = 33)	High *E/e*′ (*n* = 30)	*P*-value
Sex, male	13 (39.4)	13 (43.3)	0.751
Age, years	77.0 (69.5–82.0)	77.5 (72.5–80.0)	0.673
Body surface area, m^2^	1.48 (1.39–1.61)	1.51 (1.38–1.70)	0.693
Exercise intensity, METs	3.5 (3.1–4.9)	3.7 (3.1–4.4)	0.566
Peak workload, M	40.0 (30.0–55.0)	46.5 (37.5–50.0)	0.516
*At rest*			
SBP, mmHg	142.9 (±23.0)	134.6 (±19.3)	0.133
HR, b.p.m.	71.0 (63.0–85.8)	72.0 (58.0–82.0)	0.600
AVA, cm^2^	0.84 (±0.11)	0.81 (±0.13)	0.262
AVA index, cm^2^/m^2^	0.56 (±0.09)	0.53 (±0.09)	0.172
MPG, mmHg	23.2 (19.3–29.8)	24.6 (20.8–28.8)	0.752
LV mass index, g/m^2^	80.2 (73.7–95.5)	84.5 (74.7–107.9)	0.417
LVEDV, mL	77.7 (67.3–94.8)	77.0 (63.3–92.7)	0.863
LVESV, mL	25.0 (21.1–30.8)	25.3 (20.3–31.7)	0.287
LAV index, mL/m^2^	34.3 (26.7–43.3)	41.6 (31.1–52.6)	0.011
SV, mL	62.7 (57.1–72.4)	61.6 (49.8–71.3)	0.444
SVi, mL/m^2^	41.7 (35.5–48.4)	41.3 (32.6–45.7)	0.325
Low flow, %	6 (20.0)	10 (30.3)	0.348
CO, L/min	4.5 (3.8–5.3)	4.1 (3.7–4.9)	0.204
LVEF, %	68.1 (±4.6)	65.2 (±5.4)	0.028
E, cm/s	65.0 (57.0–80.0)	88.5 (63.0–114.8)	<0.001
*e*′, cm/s	4.8 (4.3–6.2)	4.6 (3.9–5.9)	0.261
*E/e*′	12.9 (9.6–15.4)	19.6 (13.6–22.4)	<0.001
*E/A*	0.66 (0.58–0.85)	0.75 (0.61–0.87)	0.203
Abnormal, %	24 (85.7)	18 (81.8)	
Pseudonormal, %	4 (14.3)	4 (18.2)	
MR ≥ 3°	0	1 (3.3)	0.290
SPAP, mmHg	25.3 (23.1–28.4)	27.9 (23.9–32.1)	0.085
TAPSE, mm	19.8 (17.0–21.6)	18.9 (16.0–21.4)	0.243
*During exercise*			
SBP, mmHg	186.7 (±29.9)	166.9 (±32.7)	0.016
HR, b.p.m.	117.0 (±23.1)	111.5 (±18.0)	0.294
AVA, cm^2^	0.96 (0.83–1.03)	0.86 (0.69–0.99)	0.050
AVA index, cm^2^/m^2^	0.63 (0.54–0.70)	0.57 (0.48–0.64)	0.025
MPG, mmHg	31.6 (24.8–38.4)	31.9 (25.9–42.1)	0.456
SV, mL	64.0 (57.5–80.1)	65.7 (53.6–76.2)	0.200
SVi, mL/m^2^	45.7 (±10.0)	41.7 (±9.3)	0.109
CO, L/min	7.9 (±1.7)	7.1 (±1.9)	0.079
LVEF, %	74.0 (69.9–76.8)	72.4 (67.0–75.4)	0.354
E, cm/s	126.0 (104.0–141.5)	140.0 (122.5–175.8)	0.004
*e*′, cm/s	11.9 (7.8–14.2)	7.3 (6.3–8.4)	<0.001
*E/e*′	10.7 (9.2–13.6)	18.2 (16.6–23.0)	<0.001
*E/A*	0.93 (0.77–1.05)	1.16 (0.92–1.50)	0.004
Abnormal, %	21 (75.0)	7 (31.8)	
Pseudonormal, %	7 (25.0)	15 (68.2)	
SPAP, mmHg	55.8 (±10.7)	57.3 (±10.5)	0.599
TAPSE, mm	25.1 (21.4–27.0)	24.3 (18.4–26.3)	0.052

Data are mean ± SD, median (IQR), or *n* (%).

## Discussion

The main findings of this study are as follows: (i) asymptomatic patients with low-gradient severe AS had worse prognoses than those with moderate AS with only a 40% event-free survival rate and (ii) an increase in the mitral *E/e*′ index during exercise was effective for stratifying prognosis in asymptomatic patients with low-gradient severe AS.

### Prognosis of low-gradient severe aortic stenosis

Previous studies have suggested that low-gradient severe AS might be caused by a reduced SV assessed using Doppler echocardiography, signifying an advanced stage of severe AS, compromised impaired ventricular function, and poor prognosis requiring early valve intervention.^11,18^ However, Jander *et al*.^10^ suggested that outcomes and progression rates in patients with low-gradient severe AS were comparable to those in patients with moderate AS. Contrastingly, the present study’s findings show worse prognoses in asymptomatic patients with low-gradient severe AS, potentially due to the inclusion of older and smaller patients with smaller LVs when compared with the study by Jander *et al*. Several mechanisms could account for the low gradient in severe AS despite preserved LVEF. One possible mechanism could be reduced SV even with preserved LVEF, due to concentric LV hypertrophy or impaired longitudinal LV myocardial function.

Nonetheless, lower transvalvular pressure gradients may still occur in patients with small body sizes and LV dimensions. There could be several reasons for the low gradient, even though there was a severely stenotic AVA in the presence of preserved ejection fraction (EF). One possible reason is the outcome of reduced SV, even though EF is preserved, due to decreased ventricular size and/or impaired myocardial function.^7,19^ However, even when the SV is normal, patients with smaller body sizes and LV dimensions may still show a lower transvalvular pressure gradient.^10^

### Exercise stress echocardiography for low-gradient severe aortic stenosis

Risk stratification of patients with asymptomatic AS remains controversial. Exercise stress echocardiography has emerged as an attractive risk-stratification tool for distinguishing asymptomatic patients with AS. An increase in MPG > 18–20 mmHg and an increase in SPAP > 60 mmHg during exercise have been suggested prognostic markers.^12,13^ However, the evidence remains limited, and Goublaire *et al*.^14^ reported that these parameters are ineffective for prognostic stratification. Furthermore, studies using stress echocardiography in patients with low-gradient severe AS are limited. Clavel *et al*. investigated the prognostic value of exercise and dobutamine stress echocardiography (DSE) in patients with severe PLFLG AS. They reported that prognostic stratification of patients with PLFLG severe AS was difficult using resting echocardiographic parameters, but projected AVA was effective for prognostic stratification.^20^ This study examined asymptomatic patients with ESE and symptomatic patients with DSE but did not discuss any parameters that reflect haemodynamics during exercise. It is the first study to examine ESE in patients with asymptomatic low-gradient severe AS, including echocardiographic index haemodynamics during exercise. Haemodynamic changes during exercise in patients with symptomatic, paradoxical, low-gradient severe AS have been previously studied using cardiopulmonary exercise testing combined with right heart catheterization and Doppler echocardiographic measurements. Peak oxygen consumption correlated inversely with the rate of pulmonary capillary wedge pressure (PCWP) increase. The ability to reduce vascular and valvular loads determines the effect of exercise on the PCWP. Baseline haemodynamic parameters did not predict the response to PCWP. Additionally, the haemodynamic responses did not show significant differences between patients with low flow (SVi ≤ 35 mL/m^2^) and those with the normal flow (SVi > 35 mL/m^2^).^21^

The current study investigated the relationship between prognosis and ESE parameters. MPG and EIPH,^12,13^ which were previously reported in asymptomatic AS, were difficult to use for prognostic stratification. However, the mitral *E/e*′ index during exercise was useful for prognostic stratification in patients with low-gradient severe AS. Changes in MPG and EIPH are affected by the SV and HR and may not be good prognostic indicators in low-gradient severe AS with reduced SV. In addition, it is difficult to assess whether a patient has EIPH because the right atrial pressure during exercise is difficult to estimate and is sometimes underestimated.^22^ The mitral *E/e*′ index correlates with LV stiffness and fibrosis^23,24^ and is recognized as one of the indicators of LV diastolic function. In patients with heart failure with preserved ejection fraction (HFpEF), no increase in LV filling pressure was observed at rest; however, there were cases in which there was an increase in LV filling pressure with exercise. During exercise, in patients with HFpEF, impaired early diastolic relaxation, reduced increments in suction, and poor LV compliance result in insufficient increments in SV and CO, leading to increased LV filling pressure and higher PASP.^25,26^ The mitral *E/e*′ index correlates with invasively measured LVEDP during exercise.^26^ Therefore, the mitral *E/e*′ index during exercise is an important parameter in diagnosing HFpEF.^27^ In addition, patients with elevated mitral *E/e*′ index during exercise are at increased risk of death.^28^ A previous study showed that patients with symptomatic PLFLG with severe AS had a more advanced stage of diastolic dysfunction and significantly worse prognoses than those with asymptomatic PLFLG with severe AS.^7,9^ These results suggested a possible link between severe AS and HFpEF in some patients.^29^ Based on the results of the present study, patients with asymptomatic low-gradient severe AS who exhibited an increase in *E/e*′ during exercise, which is associated with HFpEF, were observed to have poorer prognoses.

### Limitations

This study had several limitations. First, this was a single-centre, retrospective observational study, although long-term follow-up was available. Therefore, inherent bias cannot be excluded in this study type. Although the sample size was comparable to that of previous studies, it was relatively small, with a limited number of composite events. To date, no multicentre studies have been conducted on ESE in patients with low-gradient severe AS. Therefore, these results should be validated in multicentre prospective studies with larger populations. Second, the study population consisted of asymptomatic patients with moderate- or low-gradient severe AS, who could undergo exercise testing. This study does not reflect low-gradient AS as a whole because it excluded patients who underwent SAVR or TAVR within 90 days of ESE and included asymptomatic patients. However, the results may reflect actual clinical practice since ESE is contraindicated in patients with symptomatic severe AS.^30^ Third, patients with abnormal exercise test findings and early intervention were excluded, and only those who were followed up with conservative treatment were included. Therefore, the study did not reflect the overall prognoses of patients with low-grade severe AS. Fourth, most of the events in this study were AVR, although cases with AVR within 90 days of ESE were excluded. Most of the patients who underwent AVR had symptom onset and the indication was also determined by the heart-valve team. Waiting until heart failure hospitalization for AVR would be difficult in clinical practice, given the impact on patient outcomes. Fourth, this study examined both the low-flow and normal-flow groups, and there was no difference in prognosis between these groups. There are several reasons for this; for example, asymptomatic patients were entered during outpatient consultation, Japanese patients with AS are more likely to have a sigmoid-shaped septum and the Doppler SV may have been overestimated,^31,32^ Some patients were diagnosed as asymptomatic in the outpatient setting but were symptomatic in the exercise stress test, and flow statuses may have changed during follow-up.^33^ Fifth, although only 1 of 122 patients in the present cohort had mitral regurgitation of >3°, care should be taken in the interpretation of *E/e*′ and exercise stress echocardiography when significant mitral regurgitation is present. Sixth, we obtained *e*′ at the septal mitral annulus only as in a previous study. It is difficult to measure e′ at the septal and lateral mitral annulus within a limited time during exercise and correct alignment of Doppler angle tends to be difficult during exercise when measuring *e*′ at the lateral mitral annulus. Seventh, some patients with moderate AS who underwent ESE had ambiguous symptoms. Therefore, selection bias may not be eliminated in the comparison of moderate AS to low-gradient severe AS. Last, the calcium score calculated by CT, which is useful for diagnosing the severity of low-gradient AS with preserved EF, was not measured in this study. Therefore, it is not possible to verify severity in low-gradient AS.

## Conclusion

This study suggests that patients with low-gradient severe AS have worse prognoses than those with moderate AS. The mitral *E/e*′ ratio during exercise is a useful parameter for risk stratification of patients with low-gradient severe AS. Left ventricular diastolic function during exercise may play a crucial role in decision-making for patients with low-gradient severe AS.

## Data Availability

The data underlying this article were provided by St Marianna University Hospital. Data will be shared on request to the corresponding author with the permission of St Marianna University Hospital.

## Lead author biography

Daisuke Miyahara is a cardiologist at St Marianna University School of Medicine in Kawasaki, Japan. His research interests include exercise stress echocardiography, structured heart disease, chronic heart failure, and cardiomyopathies.

## References

[1] Iung B, Baron G, Butchart EG, Delahaye F, Gohlke-Bärwolf C, Levang OW, Tornos P, Vanoverschelde JL, Vermeer F, Boersma E. A prospective survey of patients with valvular heart disease in Europe: the Euro Heart Survey on Valvular Heart Disease. Eur Heart J 2003;24:1231–1243. 12831818 10.1016/s0195-668x(03)00201-x

[2] Nkomo VT, Gardin JM, Skelton TN, Gottdiener JS, Scott CG, Enriquez-Sarano M. Burden of valvular heart diseases: a population-based study. Lancet 2006;368:1005–1011. 16980116 10.1016/S0140-6736(06)69208-8

[3] Stewart BF, Siscovick D, Lind BK, Gardin JM, Gottdiener JS, Smith VE, Kitzman DW, Otto CM. Clinical factors associated with calcific aortic valve disease. Cardiovascular Health Study. J Am Coll Cardiol 1997;29:630–634. 9060903 10.1016/s0735-1097(96)00563-3

[4] Lindroos M, Kupari M, Heikkilä J, Tilvis R. Prevalence of aortic valve abnormalities in the elderly: an echocardiographic study of a random population sample. J Am Coll Cardiol 1993;21:1220–1225. 8459080 10.1016/0735-1097(93)90249-z

[5] Minners J, Allgeier M, Gohlke-Baerwolf C, Kienzle RP, Neumann FJ, Jander N. Inconsistencies of echocardiographic criteria for the grading of aortic valve stenosis. Eur Heart J 2008;29:1043–1048. 18156619 10.1093/eurheartj/ehm543

[6] Christensen KL, Ivarsen HR, Thuesen L, Kristensen B, Egeblad H. Aortic valve stenosis: fatal natural history despite normal left ventricular function and low invasive peak-to-peak pressure gradients. Cardiology 2004;102:147–151. 15334024 10.1159/000080482

[7] Hachicha Z, Dumesnil JG, Bogaty P, Pibarot P. Paradoxical low-flow, low-gradient severe aortic stenosis despite preserved ejection fraction is associated with higher afterload and reduced survival. Circulation 2007;115:2856–2864. 17533183 10.1161/CIRCULATIONAHA.106.668681

[8] Barasch E, Fan D, Chukwu EO, Han J, Passick M, Petillo F, Norales A, Reichek N. Severe isolated aortic stenosis with normal left ventricular systolic function and low transvalvular gradients: pathophysiologic and prognostic insights. J Heart Valve Dis 2008;17:81–88. 18365573

[9] Dumesnil JG, Pibarot P, Carabello B. Paradoxical low flow and/or low gradient severe aortic stenosis despite preserved left ventricular ejection fraction: implications for diagnosis and treatment. Eur Heart J 2010;31:281–289. 19737801 10.1093/eurheartj/ehp361 PMC2814220

[10] Jander N, Minners J, Holme I, Gerdts E, Boman K, Brudi P, Chambers JB, Egstrup K, Kesäniemi YA, Malbecq W, Nienaber CA, Ray S, Rossebø A, Pedersen TR, Skjærpe T, Willenheimer R, Wachtell K, Neumann F-J, Gohlke-Bärwolf C. Outcome of patients with low-gradient “severe” aortic stenosis and preserved ejection fraction. Circulation 2011;123:887–895. 21321152 10.1161/CIRCULATIONAHA.110.983510

[11] Otto CM, Nishimura RA, Bonow RO, Carabello BA, Erwin JP III, Gentile F, Jneid H, Krieger EV, Mack M, McLeod C, O’Gara PT, Rigolin VH, Sundt TM, Thompson A, Toly C. 2020 ACC/AHA guideline for the management of patients with valvular heart disease: a report of the American College of Cardiology/American Heart Association Joint Committee on Clinical Practice Guidelines. Circulation 2021;143:e72–e227. 33332150 10.1161/CIR.0000000000000923

[12] Lancellotti P, Lebois F, Simon M, Tombeux C, Chauvel C, Pierard LA. Prognostic importance of quantitative exercise Doppler echocardiography in asymptomatic valvular aortic stenosis. Circulation 2005;112:I377–I382. 16159850 10.1161/CIRCULATIONAHA.104.523274

[13] Lancellotti P, Magne J, Donal E, O'Connor K, Dulgheru R, Rosca M, Pierard LA. Determinants and prognostic significance of exercise pulmonary hypertension in asymptomatic severe aortic stenosis. Circulation 2012;126:851–859. 22832784 10.1161/CIRCULATIONAHA.111.088427

[14] Goublaire C, Melissopoulou M, Lobo D, Kubota N, Verdonk C, Cimadevilla C, Codogno I, Brochet E, Vahanian A, Messika-Zeitoun D. Prognostic value of exercise-stress echocardiography in asymptomatic patients with aortic valve stenosis. JACC Cardiovasc Imaging 2018;11:787–795. 28734909 10.1016/j.jcmg.2017.03.020

[15] Mitchell C, Rahko PS, Blauwet LA, Canaday B, Finstuen JA, Foster MC, Horton K, Ogunyankin KO, Palma RA, Velazquez EJ. Guidelines for performing a comprehensive transthoracic echocardiographic examination in adults: recommendations from the American Society of Echocardiography. J Am Soc Echocardiogr 2019;32:1–64. 30282592 10.1016/j.echo.2018.06.004

[16] Briand M, Dumesnil JG, Kadem L, Tongue AG, Rieu R, Garcia D, Pibarot P. Reduced systemic arterial compliance impacts significantly on left ventricular afterload and function in aortic stenosis: implications for diagnosis and treatment. J Am Coll Cardiol 2005;46:291–298. 16022957 10.1016/j.jacc.2004.10.081

[17] Hirasawa K, Izumo M, Suzuki K, Suzuki T, Ohara H, Watanabe M, Sato Y, Kamijima R, Nobuoka S, Akashi YJ. Value of transvalvular flow rate during exercise in asymptomatic patients with aortic stenosis. J Am Soc Echocardiogr 2020;33:438–448. 32005565 10.1016/j.echo.2019.11.007

[18] Vahanian A, Beyersdorf F, Praz F, Milojevic M, Baldus S, Bauersachs J, Capodanno D, Conradi L, De Bonis M, De Paulis R, Delgado V. 2021 ESC/EACTS guidelines for the management of valvular heart disease. Eur Heart J 2022:43:561–632. 34453165 10.1093/eurheartj/ehab395

[19] Cramariuc D, Rieck AE, Staal EM, Wachtell K, Eriksen E, Rossebø AB, Gerdts E. Factors influencing left ventricular structure and stress-corrected systolic function in men and women with asymptomatic aortic valve stenosis (a SEAS substudy). Am J Cardiol 2008;101:510–515. 18312768 10.1016/j.amjcard.2007.09.100

[20] Clavel MA, Ennezat PV, Maréchaux S, Dumesnil JG, Capoulade R, Hachicha Z, Mathieu P, Bellouin A, Bergeron S, Meimoun P, Arsenault M, Le Tourneau T, Pasquet A, Couture C, Pibarot P. Stress echocardiography to assess stenosis severity and predict outcome in patients with paradoxical low-flow, low-gradient aortic stenosis and preserved LVEF. JACC Cardiovasc Imaging 2013;6:175–183. 23489531 10.1016/j.jcmg.2012.10.015

[21] Pérez Del Villar C, Yotti R, Espinosa M, Barrio A, Lorenzo MJ, Sánchez Fernández PL, Benito Y, Prieto R, Pérez David E, Martínez-Legazpi P, Fernández-Avilés F, Bermejo J. The functional significance of paradoxical low-gradient aortic valve stenosis: hemodynamic findings during cardiopulmonary exercise testing. JACC Cardiovasc Imaging 2017;10:29–39. 27568118 10.1016/j.jcmg.2016.03.018

[22] Obokata M, Kane GC, Sorimachi H, Reddy YNV, Olson TP, Egbe AC, Melenovsky V, Borlaug BA. Noninvasive evaluation of pulmonary artery pressure during exercise: the importance of right atrial hypertension. Eur Respir J 2020;55:1901617. 31771997 10.1183/13993003.01617-2019 PMC13077671

[23] Kasner M, Westermann D, Steendijk P, Gaub R, Wilkenshoff U, Weitmann K, Hoffmann W, Poller W, Schultheiss H-P, Pauschinger M, Tschöpe C. Utility of Doppler echocardiography and tissue Doppler imaging in the estimation of diastolic function in heart failure with normal ejection fraction: a comparative Doppler-conductance catheterization study. Circulation 2007;116:637–647. 17646587 10.1161/CIRCULATIONAHA.106.661983

[24] Kasner M, Westermann D, Lopez B, Gaub R, Escher F, Kühl U, Schultheiss H-P, Tschöpe C. Diastolic tissue Doppler indexes correlate with the degree of collagen expression and cross-linking in heart failure and normal ejection fraction. J Am Coll Cardiol 2011;57:977–985. 21329845 10.1016/j.jacc.2010.10.024

[25] Erdei T, Smiseth OA, Marino P, Fraser AG. A systematic review of diastolic stress tests in heart failure with preserved ejection fraction, with proposals from the EU-FP7 MEDIA study group. Eur J Heart Fail 2014;16:1345–1361. 25393338 10.1002/ejhf.184

[26] Burgess MI, Jenkins C, Sharman JE, Marwick TH. Diastolic stress echocardiography: hemodynamic validation and clinical significance of estimation of ventricular filling pressure with exercise. J Am Coll Cardiol 2006;47:1891–1900. 16682317 10.1016/j.jacc.2006.02.042

[27] Pieske B, Tschöpe C, de Boer RA, Fraser AG, Anker SD, Donal E, Edelmann F, Fu M, Guazzi M, Lam CS, Lancellotti P. How to diagnose heart failure with preserved ejection fraction: the HFA-PEFF diagnostic algorithm: a consensus recommendation from the Heart Failure Association (HFA) of the European Society of Cardiology (ESC). Eur J Heart Fail;220:391–412. 10.1002/ejhf.1741 32133741

[28] Luong CL, Anand V, Padang R, Oh JK, Arruda-Olson AM, Bird JG, Pislaru C, Thaden JJ, Pislaru SV, Pellikka PA. Prognostic significance of elevated left ventricular filling pressures with exercise: insights from a cohort of 14,338 patients. J Am Soc Echocardiogr 2023;S0894-7317(23)00595-3. 10.1016/j.echo.2023.11.012 38000684

[29] Namisaki H, Nagata Y, Seo Y, Ishizu T, Izumo M, Akashi YJ, Yamashita E, Otsuji Y, Takeuchi M. Symptomatic paradoxical low gradient severe aortic stenosis: a possible link to heart failure with preserved ejection fraction. J Cardiol 2019;73:536–543. 30617000 10.1016/j.jjcc.2018.12.017

[30] Atterhög JH, Jonsson B, Samuelsson R. Exercise testing: a prospective study of complication rates. Am Heart J 1979;98:572–579. 495403 10.1016/0002-8703(79)90282-5

[31] Sato K, Seo Y, Ishizu T, Nakajima H, Takeguchi M, Izumo M, Suzuki K, Akashi YJ, Otsuji Y, Aonuma K. Reliability of aortic stenosis severity classified by 3-dimensional echocardiography in the prediction of cardiovascular events. Am J Cardiol 2016;118:410–417. 27287062 10.1016/j.amjcard.2016.05.016

[32] Koto D, Izumo M, Machida T, Suzuki K, Yoneyama K Y, Suzuki T, Kamijima R, Kobayashi Y, Harada T, Akashi YJ. Geometry of the left ventricular outflow tract assessed by 3D TEE in patients with aortic stenosis: impact of upper septal hypertrophy on measurements of Doppler-derived left ventricular stroke volume. J Echocardiogr 2018;16:162–172. 29797230 10.1007/s12574-018-0383-7

[33] Zusman O, Pressman GS, Banai S, Finkelstein A, Topilsky Y. Intervention versus observation in symptomatic patients with normal flow low gradient severe aortic stenosis. JACC Cardiovasc Imaging 2018;11:1225–1232. 29055632 10.1016/j.jcmg.2017.07.020

